# Quantitative analysis of in-TIPS thrombosis in abdominal CT

**DOI:** 10.1016/j.ejro.2022.100405

**Published:** 2022-02-23

**Authors:** Simon Bernatz, Inga Weitkamp, Jan-Erik Scholtz, Vitali Koch, Leon D. Grünewald, Christoph Mader, Jörg Ackermann, Moritz H. Albrecht, Simon S. Martin, Thomas J. Vogl, Scherwin Mahmoudi

**Affiliations:** a Department of Diagnostic and Interventional Radiology, Institute for Diagnostic and Interventional Radiology, University Hospital Frankfurt, Goethe University Frankfurt am Main, Theodor-Stern-Kai 7, 60590, Frankfurt am Main, Germany; b Department of Molecular Bioinformatics, Institute of Computer Science, Johann Wolfgang Goethe-University, 60325, Frankfurt am Main, Germany

**Keywords:** CT, Computed tomography, HU, Hounsfield units, IVC, Inferior vena cava, PV, Portal vein, ROI, Region of interest, TIPS, Transjugular intrahepatic portosystemic shunt, YE, Years of experience, Transjugular Intrahepatic Portosystemic Shunt, TIPS, In-TIPS thrombosis, Quantitative Imaging, CT, Angiography

## Abstract

**Purpose:**

To identify transjugular intrahepatic portosystemic shunt (TIPS) thrombosis in abdominal CT scans applying quantitative image analysis.

**Materials and methods:**

We retrospectively screened 184 patients to include 20 patients (male, 8; female, 12; mean age, 60.7 ± 8.87 years) with (case, n = 10) and without (control, n = 10) in-TIPS thrombosis who underwent clinically indicated contrast-enhanced and unenhanced abdominal CT followed by conventional TIPS-angiography between 08/2014 and 06/2020. First, images were scored visually. Second, region of interest (ROI) based quantitative measurements of CT attenuation were performed in the inferior vena cava (IVC), portal vein and in four TIPS locations. Minimum, maximum and average Hounsfield unit (HU) values were used as absolute and relative quantitative features. We analyzed the features with univariate testing.

**Results:**

Subjective scores identified in-TIPS thrombosis in contrast-enhanced scans with an accuracy of 0.667 – 0.833. Patients with in-TIPS thrombosis had significantly lower average (p < 0.001), minimum (p < 0.001) and maximum HU (p = 0.043) in contrast-enhanced images. The in-TIPS / IVC ratio in contrast-enhanced images was significantly lower in patients with in-TIPS thrombosis (p < 0.001). No significant differences were found for unenhanced images. Analyzing the visually most suspicious ROI with consecutive calculation of its ratio to the IVC, all patients with a ratio < 1 suffered from in-TIPS thrombosis (p < 0.001, sensitivity and specificity = 100%).

**Conclusion:**

Quantitative analysis of abdominal CT scans facilitates the stratification of in-TIPS thrombosis. In contrast-enhanced scans, an in-TIPS / IVC ratio < 1 could non-invasively stratify all patients with in-TIPS thrombosis.

## Highlights

• Quantitative image analyses of CT scans allow the diagnosis of in-TIPS thrombosis.
• Negative quantitative image analysis may reduce the necessity of an angiography.
• Unenhanced CT scans have no additional benefit for diagnosis of in-TIPS thrombosis.

## 1 Introduction

Chronic liver disease and liver cirrhosis are major sources of morbidity and mortality worldwide [1]. In chronic liver disease and liver cirrhosis, many of the therapeutically relevant complications arise from portal hypertension, which is primarily caused by an increased vascular resistance to portal blood flow and structural hepatic tissue alterations [2]. Portal hypertension is defined as portal venous pressure above 10 mmHg [3], [4]. Complications include variceal bleeding, portal hypertensive gastropathy, hepatic encephalopathy, and ascites [3].

Pharmacological treatment options with somatostatin, octreotid, terlipressin, and beta-blockers play a central role in prevention and therapy of portal hypertension [5], [6]. In patients with portal hypertension who do not respond to conservative therapy, interventional radiology offers a relatively low invasive alternative to reduce portal hypertension compared to open surgery [7]. In an radiologic intervention, a transjugular intrahepatic portosystemic shunt (TIPS) can be installed as an approach to connect the inflow portal vein (PV) and the outflow hepatic vein to reduce portal venous pressure and its potentially life-threatening complications [8], [9], [10]. Inevitably, complications such as TIPS dysfunction and in-TIPS thrombosis can occur and have to be diagnosed quickly and reliably [11], [12], [13].

In case of suspected TIPS dysfunction or in-TIPS thrombosis, contrast-enhanced and unenhanced computed tomography (CT) examinations of the abdomen are frequently performed to assess contrast opacification within the TIPS lumen [14]. For this purpose, the reader has to evaluate subjectively whether a TIPS dysfunction or in-TIPS thrombosis is present, and whether the current gold standard – an invasive angiography – has to be performed for further evaluation and possible intervention [15], [16]. Computational quantitative imaging with absolute and relative region of interest (ROI) based values may be feasible to develop an objective, reader-independent and more accurate approach to assess TIPS dysfunction due to in-TIPS thrombosis.

In this retrospective feasibility study, we applied subjective scores of in-TIPS thrombosis probability and quantitative image analysis techniques to assess the semi-automatic predictability of in-TIPS thrombosis in contrast-enhanced and unenhanced CT scans. We aimed at proposing a relative cut-off value to reliably and non-invasively predict in-TIPS thrombosis in contrast-enhanced and unenhanced CT scans of the abdomen.

## 2 Material and methods

### 2.1 Study design

We obtained institutional review board approval and written informed consent was waived. The patient population was not reported previously.

**Fig. 1:**
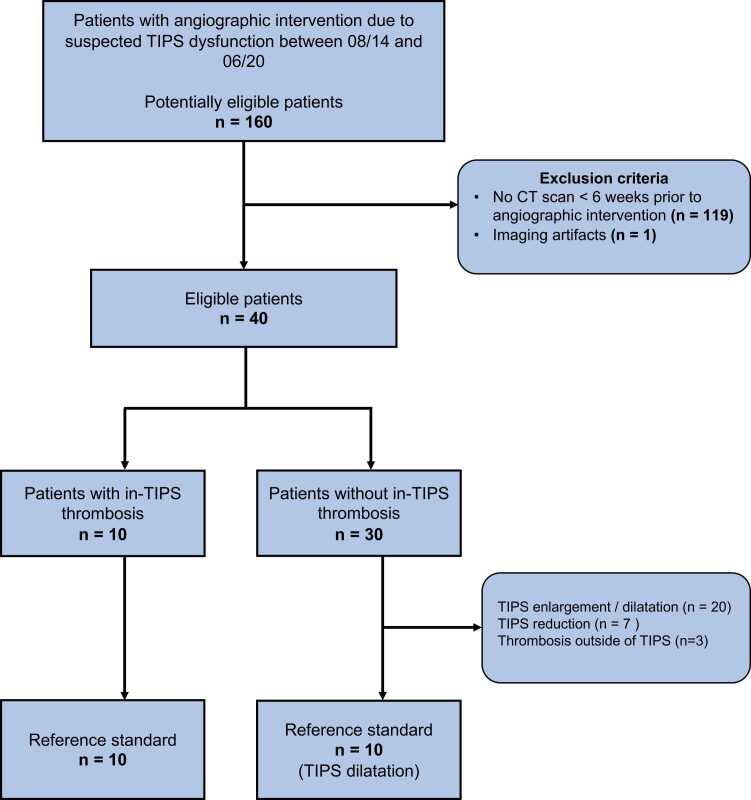
**:** STARD flowchart of patient inclusion, TIPS, transjugular intrahepatic portosystemic shunt.

We designed our study as a case-control study. We retrospectively screened 184 consecutive patients who obtained a clinically indicated angiographic examination of their TIPS between 08/2014 and 06/2020. We enrolled a final study cohort of 20 patients (in-TIPS thrombosis (case): n = 10; male, 6, female, 4; age, 62.5 (36−73) years; control cohort (control): n = 10; male, 6, female, 4; age, 62.5 (57−74) years). We stratified all patients who had angiographic intervention due to suspected TIPS dysfunction between 08/14 and 06/20. Due to a small sample size of cases, we chose a 1:1 case-control study design to match respective control cases. Inclusion criteria were (I) angiographic examination of an existing TIPS, (II) CT examination < 6 weeks prior to angiographic examination, (III) case: suspicion of in-TIPS thrombosis in the CT examination with angiographic confirmation, (IV) control: exclusion of in-TIPS thrombosis in the angiographic examination. Exclusion criteria were (I) age < 18 years, (II) imaging artifacts. The acquisition protocol included unenhanced and contrast-enhanced imaging. One patient of the case-cohort did not obtain an unenhanced acquisition. Fig. 1 depicts the detailed flowchart of patient inclusion.

### 2.2 CT imaging acquisition and examination

**Fig. 2:**
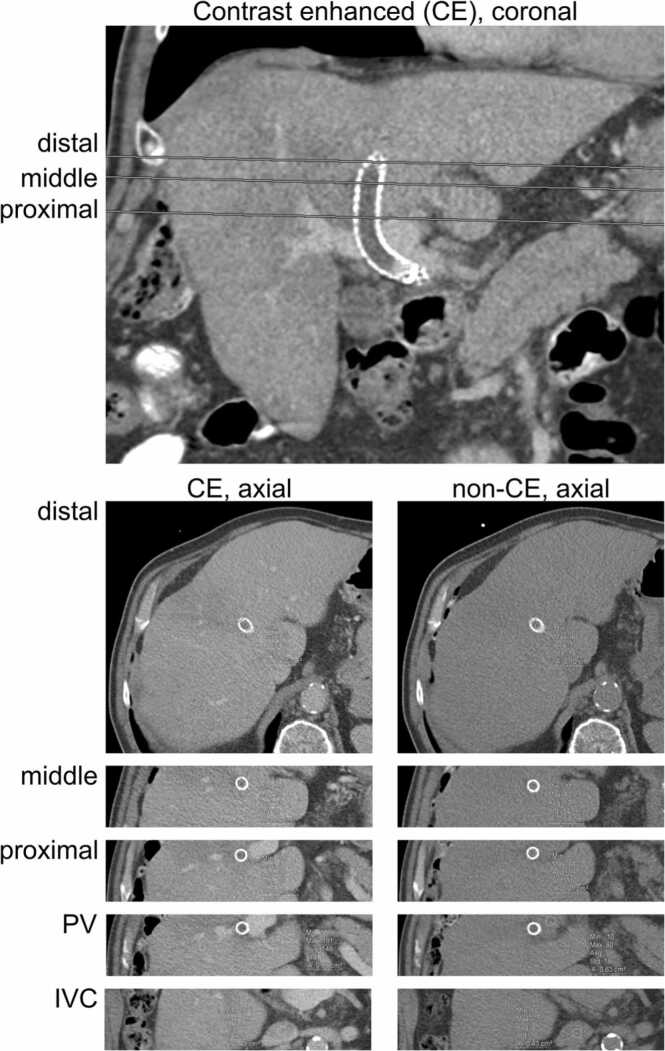
**:** Example of representative region of interest (ROI) placement. Contrast-enhanced (CE) and unenhanced computed tomography examination of the abdomen of a patient with a thrombus within the lumen of the transjugular intrahepatic portosystemic shunt (TIPS). The coronal plane depicts the respective levels of axial ROI-placement within the TIPS lumen. In this patient, all three axial planes represented areas of highest suspicion of in-TIPS thrombosis, therefore an additional ROI of highest suspicion is not depicted. Respective intraluminal regions used to measure the portal vein (PV) and inferior vena cava (IVC) are depicted. Quantitative measurements consisted of minimum, maximum, average, standard deviation and area.

The patients received a clinically indicated CT scan of the abdomen without (n = 19) and with (n = 20) contrast-enhanced acquisition. All patients, except one, were examined in domo. The acquisition protocol operated the x-ray tube at 127.89 ± 17.82 kV, 149 ± 64.07 mAs (unenhanced) and 135.50 ± 17.31 kV, 141.85 ± 78.05 mAs (contrast-enhanced). For the unenhanced and contrast-enhanced acquisition, we obtained a mean volume CT dose index of 11.54 ± 4.64 mGy, 12.38 ± 4.67 mGy and a mean dose-length product of 570.05 ± 278.85 mGy × cm, 603.92 ± 281.10 mGy × cm. We performed the subjective and ROI-based analysis employing image series which were reconstructed in axial plane with 5 mm slice thickness. For the subjective analysis, three independent readers (IW, SB, SM) scored their individual level of agreement with the diagnosis of in-TIPS thrombosis using a five-point Likert-scale (1, strongly disagree; 2, disagree; 3, unclear; 4, agree; strongly agree). We calculated the intra-class correlation coefficients (ICC) to assess the inter-reader agreement applying ICC3 of the Pingouin package [17] in Python. We independently drew 70% of the data for training and 30% for testing of a logistic regression model (scikit-learn [18]) to predict in-TIPS thrombosis based on the subjective scores of each reader. For the quantitative analysis, we manually drew ROIs in three representative axial planes within the TIPS lumen (proximal, middle, distal) and within the visually most suspicious area for in-TIPS thrombosis, sparing border zones to reduce partial volume artifacts. We drew ROIs in the lumen of the PV and inferior vena cava (IVC) as reference values. We depict the systematic ROI definition in Fig. 2. ROI circumscription was performed by one investigator (IW, 1 year of experience (YE)), blinded to the written reports and patient characteristics. ROIs were reviewed by a second blinded, independent reader (SM with 2.5 YE, in training). ROIs with disagreement were re-reviewed by a third blinded reader (SB, 3 YE, in training) to obtain final consensus agreement. We performed quantitative analysis employing ROI-measurements on dedicated workstations (Centricity Universal Viewer, version 7, GE Healthcare).

### 2.3 Reference standard

Ground-truth with confirmation or exclusion of in-TIPS thrombosis was based on the angiographic intervention.

### 2.4 Evaluation approach and statistical analysis

We performed standard descriptive statistics and graphical illustrations employing JMP 14 (SAS) and Affinity Designer 1.8.5.703 (Serif (Europe) Ltd). Further analysis was done in Python. Detailed information about experimental protocols and statistical tests are given in the legends of the tables and figures.

## 3 Results

### 3.1 Study population

Employing a case-control study design we analyzed 20 patients (case: n = 10; male, 6; female, 4; age, 62.5 (36−73) years; control cohort (control): n = 10; male, 6; female, 4; age, 62.5 (57−74) years). The case-control cohorts did not differ in sex, age, period since TIPS-intervention, and timespan between CT acquisition and angiographic intervention. No inter-cohort difference was found comparing the size of the employed ROI and standard deviation. We depict detailed patient characteristics in Table 1.

**Table 1:** Clinical and epidemiological characteristics of included patients.

variable	study cohort
patients (n)	20
in-TIPS thrombus, CE / UE	control cohort, CE / UE
10 / 9 [NA: 1]	10 / 10
median age (y)	
diagnosis of thrombus/ CT examination	62.5 (36 – 73)
control cohort CT examination	62.5 (57 – 74)
sex	
in-TIPS thrombus (male / female)	6 / 4
control cohort (male / female)	6 / 4
median time (d), CT to angiography	
in-TIPS thrombus	3 (0 – 36)
control cohort	2 (0 – 26)
median time (m) since TIPS-procedure	
in-TIPS thrombus	15.5 (1 – 166) [NA: 4]
control cohort	1 (0 – 43) [NA: 3]
Indication for angiography (n)	
in-TIPS thrombus	
dysfunction by thrombus	10
control cohort	
TIPS extension	8
TIPS diameter reduction	2
CT scanner (n)	
in-TIPS thrombus	
CT-F	5
CT-D	4
ex domo	1
control cohort	
CT-F	10
Region of interest, size (cm²) #	
in-TIPS thrombus, CE / UE	0.127 (0.060/0.230) / 0.123 (0.053/0.237)
Control cohort, CE / UE	0.098 (0.050/0.140) / 0.100 (0.050/0.143)
Standard Deviation #	
in-TIPS thrombus, CE / UE	18.833 (7.667/38.333) / 14.333 (8.667/22.667)
Control cohort, CE / UE	11 (6.000/24.000) / 12.167 (6.667/36.667)

If not otherwise depicted, the numbers without parenthesis depict absolute numbers. Data in round parenthesis are the min/max values (interquartile range); # median of the average mean values of proximal, middle, distal region of interest with min/max in parenthesis. Data in square parenthesis are not available values, excluded in the analysis. For statistical analysis, groups were compared using two-sided student’s t-test or Likelihood Ratio/ Pearson Test. CE, contrast enhanced; CT-D, CT SOMATOM Definition AS; CT-F, CT SOMATOM Force; d, days; m, months; NA, not available; TIPS, transjugular intrahepatic portosystemic shunt; UE, unenhanced; y, years.

### 3.2 Subjective image analysis revealed a border zone of ambiguity

**Fig. 3:**
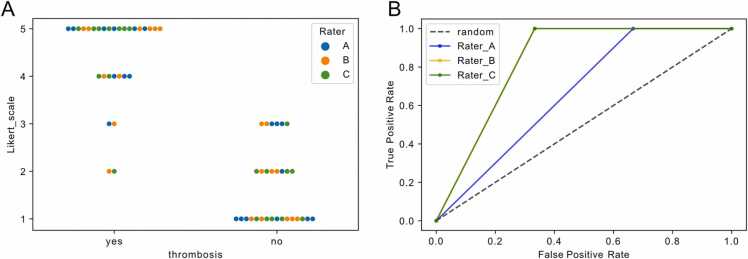
**:** Visual scoring of diagnostic accuracy In A) the individual scores of each rater are shown in a swarmplot. In B) the receiver operating characteristics (ROC) curve is shown for each rater using a logistic regression model. Rater B and C showed equivalent results in the model and the respective ROC curve are overlying, respectively.

The subjective score revealed high inter-reader robustness (ICC3 = 0.944) and the majority of subjective scores were congruent with the diagnosis or exclusion of in-TIPS thrombosis (Fig. 3A). 13.3% (4/30) and 20% (6/30) of ratings revealed unclear subjective scores for and against the diagnosis of in-TIPS thrombosis, respectively (Fig. 3A). A logistic regression model was built using the scores of each rater which showed a diagnostic accuracy for in-TIPS thrombosis of 0.67, 0.83 and 0.83 for the individual raters (Fig. 3B).

### 3.3 Mean quantitative measurements in contrast-enhanced images differed between the cohorts

**Fig. 4:**
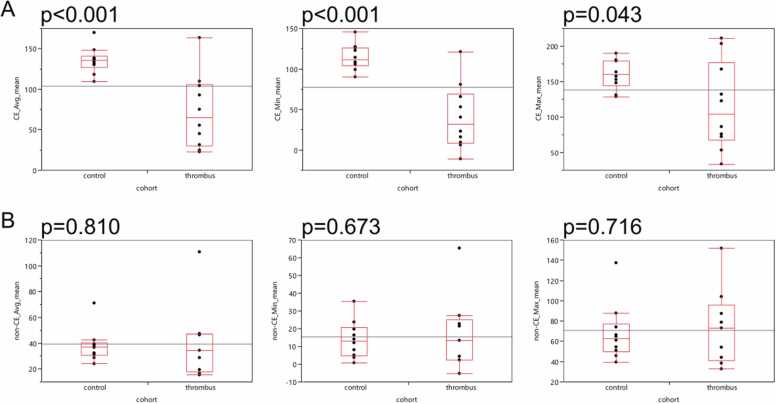
**:** Patients with in-TIPS thrombosis show variant quantitative features in contrast-enhanced images but not in unenhanced images. Box-Whisker plots for the quantitative imaging features average (avg), minimum (min), maximum (max) are shown for the mean values of the three regions of interest (ROIs) within the lumen of the transjugular intrahepatic portosystemic shunt (TIPS). In A) contrast-enhanced (CE) acquisition was performed. B) visualizes the results of the non-contrast-enhanced (non-CE) acquisition.

Second, we analyzed the mean values of the three independent ROI-based measurements (proximal, middle, distal) for the measurements of average (avg), minimum (min) and maximum (max) Hounsfield unit (HU). Employing contrast-enhanced images, patients with in-TIPS thrombosis revealed significantly lower avg HU (p < 0.001), min HU (p < 0.001) and max HU (p = 0.043) (Fig. 4A). We did not find any differences analyzing CT images of unenhanced acquisition (Fig. 4B).

### 3.4 Normalization of quantitative measurements to the inferior vena cava and portal vein

**Fig. 5:**
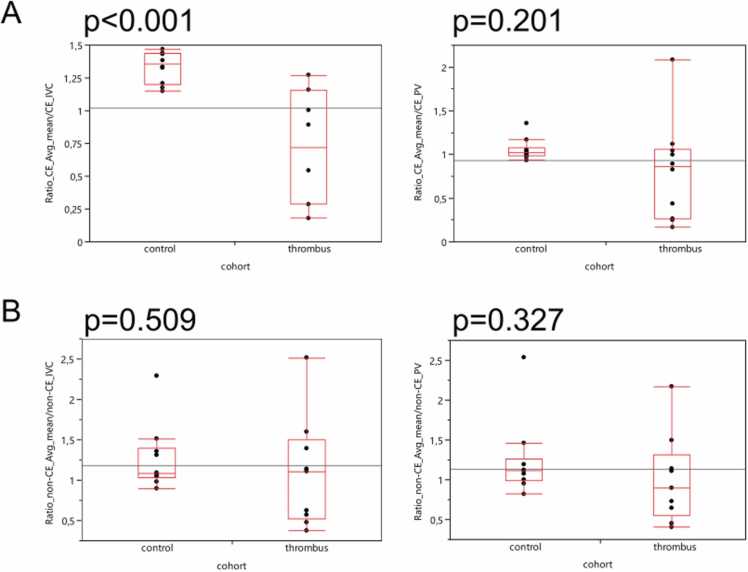
**:** Calculation of in-TIPS measurements ratio to intraluminal inferior vena cava. Box-Whisker plots for the ratios of quantitative imaging feature average (avg) for the mean values of the three regions of interest (ROIs) within the lumen of the transjugular intrahepatic portosystemic shunt (TIPS) versus an intraluminal ROI within the inferior vena cava (IVC) and portal vein (PV) are shown. In A) contrast-enhanced (CE) acquisition was performed. B) visualizes the results of the non-contrast-enhanced (non-CE) acquisition.

We computed the ratio of mean avg HU (mean value of proximal, middle, distal ROI) and avg ROI measurements within the lumen of the IVC or PV. In contrast-enhanced images, patients with in-TIPS thrombosis revealed significantly lower values for the ratio of avg HU / IVC (p < 0.001) whereas no significance was found for the ratio of avg HU / PV (p = 0.201) (Fig. 5A). Respective ratios did not yield significant differences analyzing unenhanced images (Fig. 5B).

### 3.5 Quantitative measurement of the region with highest visual suspicion of thrombus

**Fig. 6:**
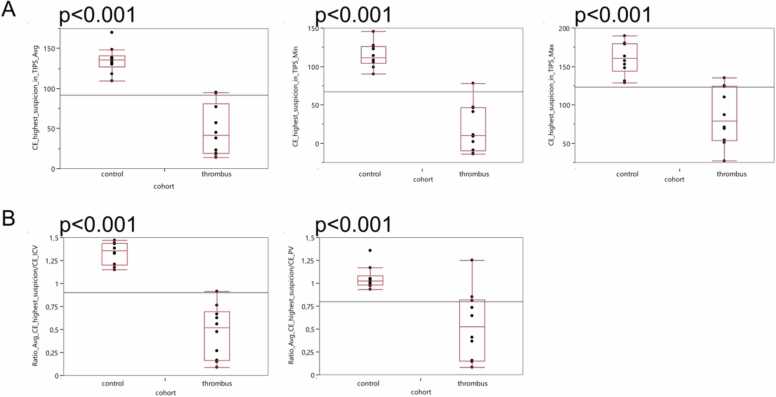
**:** Measurements of the region of interest (ROI) with the highest visual suspicion of in-TIPS thrombosis. Box-Whisker plots for the quantitative imaging features average (avg), minimum (min), maximum (max) are shown for the ROI within the lumen of the transjugular intrahepatic portosystemic shunt (TIPS) with highest visual suspicion of thrombosis, A). In B) the respective ratios of avg with ROI measurement within the inferior vena cava (IVC) and portal vein (PV) are shown. Contrast-enhanced (CE) acquisition was performed.

We measured the area within the TIPS with highest visual suspicion of thrombus. Absolute measurements of avg, min and max HU were lower for the thrombus cohort (p < 0.001; Fig. 6A). Next, we calculated the respective ratio of avg HU / ICV and avg HU / PV for the respective region of highest suspicion for thrombus. Patients with thrombus had significantly lower values (p < 0.001). Employing the ratio of avg HU / IVC, all patients with a ratio < 1 were found to suffer from in-TIPS thrombosis (Fig. 6B).

## 4 Discussion

In this study, we analyzed the computationally quantifiable differences of in-TIPS thrombosis in contrast-enhanced and unenhanced CT scans of the abdomen. We assumed that a dedicated cut-off value based on quantitative image analysis techniques may facilitate the stratification of patients with in-TIPS thrombosis in contrast-enhanced and unenhanced CT scans of the abdomen. Examining 20 CT scans, we could demonstrate that a ratio of “visually most suspicious area for in-TIPS thrombus” / IVC < 1 could non-invasively stratify all patients with in-TIPS thrombosis in contrast-enhanced CT scans of the abdomen. The quantitative approach was superior to the subjective visual scoring of in-TIPS thrombosis. We could show that unenhanced CT scans did not yield any potential to confirm or exclude in-TIPS thrombosis. Based on our findings, we conclude that quantitative image analysis of contrast-enhanced CT scans of the abdomen can objectively identify in-TIPS thrombosis in routine contrast-enhanced CT scans of the abdomen. Consequently, negative quantitative image analysis may have the potential to replace the invasive and time-consuming current diagnostic gold standard, an angiography, if no therapeutic intervention is necessary. Further, our study demonstrates that unenhanced CT scans do not inherit additional diagnostic information regarding the assessment of in-TIPS thrombosis. In cases of suspected in-TIPS thrombosis, unenhanced scans should not be routinely acquired, and radiation exposure could be reduced.

Over recent years, TIPS has gained increased acceptance in the prevention and treatment of portal hypertension and its complications [19]. Despite scientific progress and advancement in stent technology, in-TIPS thrombosis causing stent occlusion is still one of the leading complications that can lead to TIPS dysfunction [20], [21]. Since diagnostic performance of Doppler-ultrasonography has been described as poor in literature [22], [23], contrast-enhanced CT is an important alternative in the diagnostic of in-TIPS thrombosis and TIPS dysfunction [14], [24]. In addition, unlike the current angiographic gold standard, CT can be performed quickly and easily and offers a non-invasive method for the diagnosis of in-TIPS thrombosis [24], [25].

According to the triad of Virchow, there are three categories of factors that contribute to thrombosis: endothelial injury, hypercoagulability and stasis [26].

Endothelial injury is an important contributing factor in the development of thrombosis and can result from atherosclerotic disease [27], [28]. Especially in cardiac imaging, CT is a well-validated imaging modality for the assessment of atherosclerosis [29], [30].

In terms of hypercoagulability and blood constituents, several studies have investigated the potential of quantitative measurements of CT density to quantify blood components. For example, correlation of attenuation measurements in CT scans and blood components such as hemoglobin and hematocrit has been demonstrated [31], [32].

For the evaluation of stasis-associated vascular pathologies including thrombosis and embolism, contrast-enhanced CT is a common diagnostic imaging modality [33], [34]. Although application of contrast media is a standard procedure for the detection of vascular-associated pathologies, several studies revealed the value of non-contrast CT scans for the assessment of thromboembolism through detection of the hyperdense lumen sign in cases of pulmonary embolism and acute ischemic stroke [35], [36], [37]. However, in our cohort, unenhanced CT scans did not yield any potential to verify or exclude in-TIPS thrombosis. This could be explained by the fact that in cases with implanted TIPS, metal artifacts may overlay hyperdense lumen and consecutively complicate the detection of in-TIPS thrombosis [38].

In order to improve the quality and accuracy of diagnostic CT reports, an objective and reader-independent approach is essential. In an article published in 2018, the authors suggest that datafication and quantification are major elements to standardize and structure radiology reports for the purpose of quality improvement [39]. By proposing a cut-off value based on quantitative analysis techniques, our results contribute to a more objective, reader-independent approach which is a major advantage compared to examiner-dependent alternatives such as Doppler-ultrasonography.

Our study has limitations that warrant discussion. First, our feasibility study was limited to twenty patients. A bigger cohort may have been favorable. Second, conducting a retrospective study, we cannot rule out selection bias. At last, one patient was examined ex domo. Due to the small patient cohort, this patient was yet included and interscanner variability may have occurred.

## 5 Conclusions

In conclusion, this study demonstrates that quantitative image analysis techniques in contrast-enhanced CT scans can facilitate the stratification of patients with in-TIPS thrombosis. In contrast-enhanced CT scans of the abdomen, a mean HU in-TIPS / IVC ratio < 1 could non-invasively predict all patients with in-TIPS thrombosis. Invasive workup of selected cases may be avoided or direct application of wires with higher levels of stiffness may be promoted.

## Ethical approval

We obtained institutional review board (IRB) approval of the Ethical Committee at the University Hospital Frankfurt (project-number: 20/689) and written informed consent was waived for this retrospective study. The patient population was not reported previously.

## Funding statement

This research did not receive any specific grant from funding agencies in the public, commercial, or not-for-profit sectors.

## CRediT authorship contribution statement

**Simon Bernatz:** Conceptualization, Data curation, Formal analysis, Investigation, Methodology, Project administration, Software, Supervision, Validation, Writing – original draft, Writing – review & editing, **Inga Weitkamp:** Data curation, Formal analysis, Investigation, Methodology, Validation, Visualization, Writing – review & editing, **Jan-Erik Scholtz:** Data curation, Formal analysis, Investigation, Methodology, Project administration, Software, Supervision, Validation, Visualization, Writing – review & editing, **Vitali Koch:** Data curation, Formal analysis, Investigation, Methodology, Project administration, Validation, Writing – review & editing, **Leon D. Grünewald:** Data curation, Formal analysis, Investigation, Methodology, Project administration, Validation, Writing – review & editing, **Christoph Mader:** Data curation, Formal analysis, Investigation, Methodology, Software, Validation, Writing – review & editing, **Jörg Ackermann:** Conceptualization, Data curation, Formal analysis, Methodology, Software, Validation, Writing – original draft, Writing – review & editing, **Moritz H. Albrecht:** Formal analysis, Investigation, Methodology, Software, Supervision, Validation, Writing – review & editing, **Simon S. Martin:** Data curation, Formal analysis, Investigation, Methodology, Software, Supervision, Validation, Writing – review & editing, **Thomas J. Vogl:** Formal analysis, Investigation, Methodology, Project administration, Resources, Software, Supervision, Validation, Visualization, Writing – review & editing, **Scherwin Mahmoudi:** Conceptualization, Data curation, Formal analysis, Investigation, Methodology, Project administration, Software, Supervision, Validation, Visualization, Writing – original draft, Writing – review & editing.

## Declaration of Competing Interest

Moritz H. Albrecht received speaker fees from Siemens and Bracco, no conflict of interest related to the current study. The authors declare that they have no known competing financial interests or personal relationships that could have appeared to influence the work reported in this paper.
